# GLP-1-Mediated Pregnancy and Neonatal Complications in Mice

**DOI:** 10.3390/jdb13030029

**Published:** 2025-08-15

**Authors:** Rajalakshmi Ramamoorthy, Arianna K. Carden, Hussain Hussain, Brian Z. Druyan, Ping Ping Chen, Rima Hajjar, Carmen Fernandez, Nila Elumalai, Amirah B. Rashed, Karen Young, Anna Rosa Speciale, Emily M. West, Staci Marbin, Bradley Safro, Ian J. Bishop, Arumugam R. Jayakumar, Luis Sanchez-Ramos, Michael J. Paidas

**Affiliations:** 1 Department of Obstetrics, Gynecology and Reproductive Sciences, University of Miami Miller School of Medicine, Miami, FL 33136, USA; rxr1310@med.miami.edu (R.R.); bzd2@med.miami.edu (B.Z.D.); rima.hajjar@jhsmiami.org (R.H.); carmen.fernandez@jhsmiami.org (C.F.); nilanandhie@gmail.com (N.E.); arashed@med.miami.edu (A.B.R.); emw123@miami.edu (E.M.W.); sjm53@miami.edu (S.M.); bjs246@med.miami.edu (B.S.); ijbishop@med.miami.edu (I.J.B.); 2 School of Nursing and Health Studies, University of Miami, Coral Cables, Miami, FL 33146, USA; akc104@miami.edu; 3 HCA Florida Kendall Hospital, Department of Internal Medicine, Miami, FL 33175, USA; hussainhussainmd77@gmail.com; 4 Department of Pediatrics, University of Miami Miller School of Medicine, Miami, FL 33136, USA; pchen2@med.miami.edu (P.P.C.); kyoung3@med.miami.edu (K.Y.); 5 Department of Health Sciences, Obstetrics and Gynecology Branch, University of Florence, Largo Brambilla, 50134 Florence, Italy; 6 Department of Obstetrics and Gynecology, University of Florida College of Medicine, Jacksonville, FL 32209, USA; luis.sanchez@jax.ufl.edu

**Keywords:** A/J mice, gene sequencing, Glucagon-like peptide 1, neonatal complications, pregnancy

## Abstract

Glucagon-like peptide 1 (GLP-1), a hormone derived from the proglucagon gene, regulates various physiological processes; however, its impact on pregnancy outcomes remains poorly understood. Assessing the effects of GLP-1 on neonates is vital as GLP-1 is increasingly administered during pregnancy. This study evaluates the effect of GLP-1 exposure on maternal complications and neonatal defects in mice. Pregnant female A/J mice received subcutaneous injections of recombinant GLP-1 (rGLP-1; 1000 nmol/kg) on embryonic day 1 (EP, early pregnancy) or day 15 (E15, late pregnancy). Maternal and neonatal body weights, morphology, and mortality were recorded, and mRNA sequencing was conducted to analyze gene expression in neonatal tissues. Maternal body weight decreased following rGLP-1 exposure, and pups born to both the early and late exposure groups experienced significant weight loss. Pups in the late exposure group exhibited uniform skin detachment and a dramatically higher mortality rate than those born to the early exposure group. Further, RT-PCR analysis confirms the significantly increased expression of selected genes in the skin and associated pathogenesis. RNA sequencing of pups’ skin, brain, lung, and liver tissues from the late exposure group showed altered gene expression. Since maternal weight loss, increased neonatal mortality, and altered gene expression have been observed, GLP-1 receptor agonists (GLP-1RAs) should be avoided during pregnancy.

## 1. Introduction

GLP-1 receptor agonists (GLP-1RAs) have rapidly increased as a treatment for type 2 diabetes mellitus and obesity due to their effectiveness in improving glycemic control, promoting weight loss, and offering cardiovascular benefits [1]. As these medications become more commonly prescribed, particularly among women of reproductive age, concerns have arisen regarding their safety during pregnancy [2,3]. Given the critical role of GLP-1 in various physiological processes, including its presence in organs such as the pancreas, gastrointestinal tract, and brain, understanding its effects on fetal development has become increasingly important [4].

Current guidelines recommend discontinuing GLP-1RAs before conception and during pregnancy, citing potential risks to the fetus, including congenital malformations, growth defects, and adverse pregnancy outcomes [5,6,7,8]. While some studies report no increased risk of congenital malformations [2,3], other studies highlight specific organ systems, such as the cardiovascular, central nervous, and gastrointestinal systems, as particularly vulnerable to GLP-1RA exposure [4]. Given the growing use of GLP-1RAs and the uncertainties surrounding their safety during pregnancy, this study investigates the effects of recombinant GLP-1 (rGLP-1) administration during pregnancy using mice.

We examined maternal and fetal weight outcomes following exposure to rGLP-1 during early and late gestation, focusing on the molecular changes in offspring organs such as the skin, liver, lung, and brain. By isolating the effects of recombinant GLP-1, this research aims to provide clearer insights into the potential risks and benefits of GLP-1 exposure during pregnancy.

## 2. Materials and Methods

### 2.1. Sample Collection

Female A/J mice (8 weeks old, weighing 19–20 g) were obtained from Jackson Laboratory (Bar Harbor, ME, USA). All mice were housed at the University of Miami, Miller School of Medicine animal facility, in a pathogen-free environment under controlled conditions. The room temperature was maintained at 22 °C ± 1 °C with 55% ± 5% relative humidity. Mice were kept on a 12 h light/dark cycle and provided ad libitum access to food and water. The diet consisted of irradiated animal chows (Envigo, 2918, Teklad, Dublin, VA, USA) and autoclaved tap water.

Female mice (*n* = 20) were randomly selected for mating with male mice (*n* = 10) at a 2:1 female-to-male ratio. Mating pairs were housed together in a single cage for one week. Pregnancy was confirmed by the presence of a vaginal plug, which was recorded as E0 (embryonic day 0). Pregnant mice (*n* = 12) were selected for the study, according to guidelines established by the University of Miami Institutional Animal Care and Use Committee (IACUC protocol #23-162). The 12 pregnant mice were randomly divided into the following four experimental groups, with 3 pregnant mice per group: (1) Early Pregnancy (EP) rGLP-1 exposure, (2) Control for EP, (3) Late Pregnancy (LP) rGLP-1 exposure, (4) Control for LP.

Mice in the early pregnancy group received one-time subcutaneous injection of 70 μL of rGLP-1 (Cat No #G3265, Sigma Aldrich Inc., St. Louis, MO, USA) at a dose of 1000 nmol/kg body weight on day 1 of gestation (E1). Similarly, the late pregnancy group received the matching dose of rGLP-1 (1000 nmol/kg body weight) via one-time subcutaneous injection on day 15 of gestation (E15). The rGLP-1 concentration (1000 nmol/kg b.wt.) used in this study was selected based on earlier findings where body weight reduction was observed when six-week-old db/db mice (a mouse model of type 2 diabetes and obesity), adult male C57BL/6 mice, and adult male Sprague-Dawley rats were injected subcutaneously [9,10]. Early and late control groups received 70 μL of phosphate-buffered saline (PBS) on days 1 or 15. Upon completion of the treatment, brain, skin, liver, and lung tissues were collected and immediately stored at −80 °C for further analysis.

### 2.2. mRNA Sequencing

mRNA was isolated from the pup tissues collected from control (5 pups) and late GLP-1 exposure (5 pups) groups using the RNeasy micro kit (Cat #74004, Qiagen, Germantown, MD, USA). The concentration of the isolated mRNA was determined using a NanoDrop OneC instrument (Thermo Fisher Scientific, Waltham, MA, USA). mRNA quality was assessed using a High-Sensitivity RNA Analysis Kit (Cat #Q33221, Qiagen, Germantown, MD, USA). In this study, mRNA having RNA integrity number (RIN) values above 7 was considered excellent and used for sequencing. Samples are eliminated from the sequence if the RIN values are less than 7. For skin tissue, mRNA libraries were prepared using the Illumina Stranded mRNA Prep Ligation Kit (Cat #20040534, Illumina, San Diego, CA, USA, and the resulting libraries were quantified with the Qubit dsDNA Assay Kit (Cat #Q35853, Qiagen, MD, USA). Each library was acquired at a concentration of 10 nM/10 μL, followed by pooling and denaturation. The final library concentration was adjusted to 1.4 pM and sequenced on the Illumina MiniSeq platform using the High Output Kit. The sequencing configuration consisted of 150-cycle paired-end reads per sample, with a 5% PhiX spike-in control. For brain, lung, and liver tissues, mRNA library constructions were performed using the NEBNext Ultra II directional RNA library prep kit (New England Biolabs Inc., Ipswich, MA, USA). These libraries were then pooled and sequenced on the Illumina NovaseqX plus platform with paired end reads configuration.

### 2.3. Bioinformatics Analysis

Sequence alignment, normalization, and differential gene expression of skin were performed against the mouse genome version mm10 using BaseSpace software version 4.3.7 (Illumina). BaseSpace Dragon RNA and Dragon differential gene expression analysis tools were utilized to convert the sequence reads into mRNA data, followed by differential gene expression analyses. Gene Ontology (GO) and Kyoto Encyclopedia of Genes and Genomes (KEGG) pathway analysis were conducted using SR plot bioinformatics software (Beta version) [11]. Bioinformatic analyses of brain, liver, and lung tissues were performed using DESeq2 software (version 1.38.0). Gene ontology analysis was conducted using the cluster Profiler package in R programming software (version 4.4.0).

### 2.4. Real-Time PCR

A total of 2 μg/sample was subjected to cDNA synthesis using a cDNA reverse transcription kit (Cat # 4368814, Applied Biosystems, Waltham, MA, USA) according to the manufacturer’s protocol. Significantly differentially expressed genes were selected from the dataset, and primers were identified from the primer bank (see Table 1 for the list of primers). The primers were purchased from Oligos, Sigma Aldrich (Burlington, MA, USA). Quantitative real-time PCR was performed using CFX Opus 96 Real-Time PCR system (Bio-Rad Laboratories, Hercules, CA, USA) with the threshold cycle number determined by CFX Opus 96 Real-Time PCR software version. All the reactions were carried out in triplicates and the 131 threshold values were calculated and normalized to the average CT value of β-actin.

### 2.5. Statistical Analysis

Statistical analyses of the mother were performed using GraphPad Prism software (version 10.1.2), including One-way ANOVA and Tukey’s multiple comparisons test. The pup weight, survival, and Real-Time PCR data were analyzed using a t-test in GraphPad 10.1.2. *p*-values less than 0.05 were considered statistically significant.

## 3. Results

### 3.1. Morphological Changes

**Figure 1:**
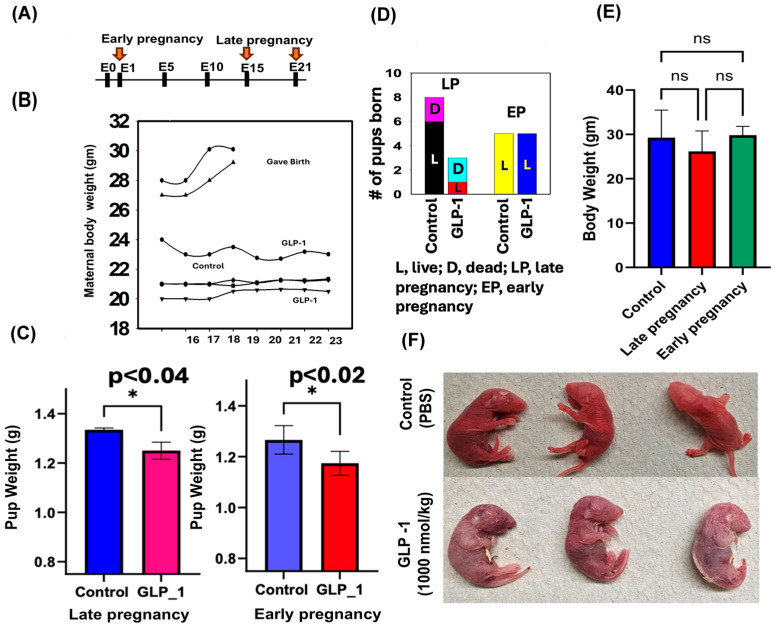
Effects of rGLP-1 exposure in early pregnancy (day 1) and late pregnancy (day 15) on maternal and pup outcomes. (**A**) Experimental timeline showing rGLP-1 exposure on day 1 in the early pregnancy group and day 15 in the late pregnancy exposure group (**B**). Maternal body weight trajectories. In the late pregnancy (LP) group (top two lines), body weight increases steadily after day 15 of rGLP-1 exposure, consistent with normal gestational weight gain. In contrast, early pregnancy exposure (third line from the top and bottom line) resulted in significantly lower and more unstable maternal body weights, while control group weights (fourth line from the top) remained relatively consistent following day 15. (**C**) Pup body weights at birth were significantly reduced in both early and late rGLP-1 exposure groups compared to controls (* *p* ≤ 0.05; *p* < 0.02 for early; *p* < 0.04 for late), indicating fetal growth restriction. (**D**) Pup mortality was much higher in the late rGLP-1 exposure group, but not significantly different in the early exposure group, suggesting increased perinatal risk from late gestational exposure. (**E**) Maternal body weight assessed after 4 months post-exposure showed a persistent reduction in the late rGLP-1 group, suggesting long-term metabolic effects of late gestational GLP-1 administration (ns-nonsignificant). (**F**) Morphological abnormalities were observed in late pregnancy (day 15) pups, including detached skin at birth.

Analysis revealed that the body weight of pregnant mice was significantly reduced after rGLP-1 exposure. Similarly, both groups of pups exposed to rGLP, early pregnancy (E1) and late pregnancy (E15), showed weight loss compared to their respective control groups (*p* < 0.02 for early pregnancy, *p* < 0.04 for late pregnancy) (Figure 1B,C). Additionally, 66% of pups born to rGLP-1-treated mothers in the late pregnancy group died, while no deaths were recorded in pups from the early pregnancy group (Figure 1D). When maternal weight was rechecked after 4 months of rGLP-1 exposure, a 13% weight decrease was observed only in the late pregnancy group mothers compared to early pregnancy mothers and control group (Figure 1E). Pups from the late rGLP-1 exposure group died within 12 h post-birth and exhibited widespread skin detachment compared to the control pups (Figure 1F). In contrast, no gross morphological abnormalities were observed in pups from the early pregnancy group.

#### 3.1.1. Molecular Changes in the Skin

**Figure 2:**
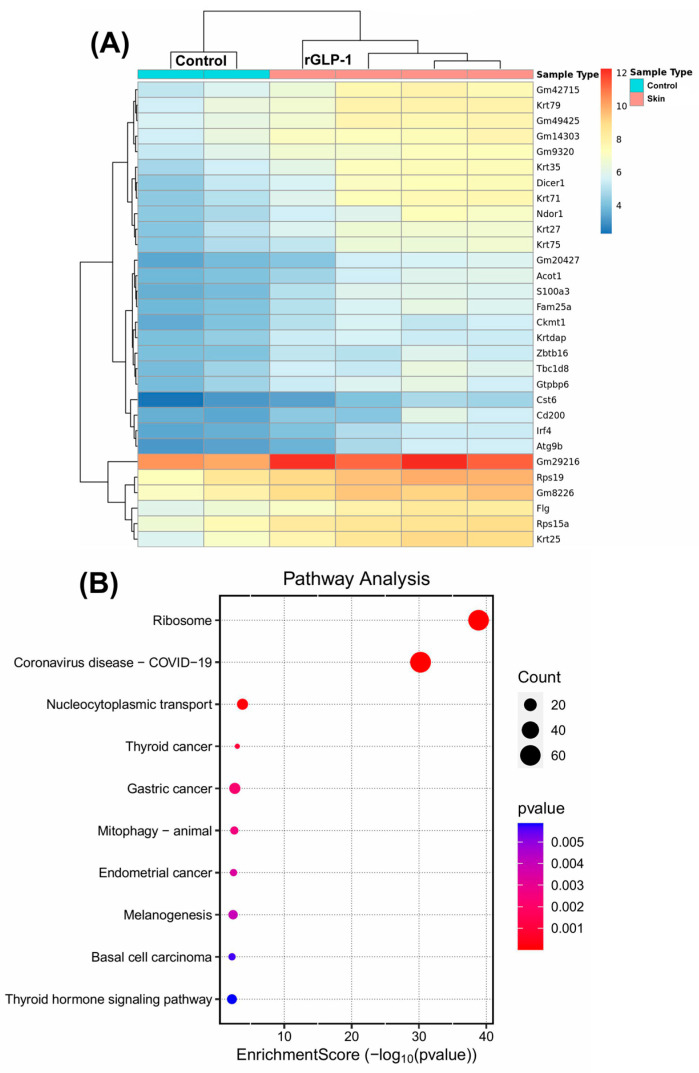
Skin gene expression profile of pups born to rGLP-1-exposed mother. (**A**) The heatmap shows 30 differentially expressed genes in the skin of pups born to rGLP-1-exposed mothers. (**B**) KEGG analysis indicates pathways related to differentially expressed genes.

Since skin changes were the primary morphological findings in pups born to rGLP-1-injected mice, we first focused on examining the skin tissue. A sequence analysis of the skin revealed a total of 1548 differentially expressed genes. Of these, 1342 genes were upregulated, and 206 genes were downregulated. A heatmap of the data illustrates the 30 most significantly upregulated genes in the skin **(**Figure 2A). Among these upregulated genes, several keratin (KRT) genes, including KRT79, KRT75, KRT71, KRT35, KRT27, KRT25, and KRTDap were significantly increased in the neonate skin. These keratin genes are crucial for the structural integrity of the skin, and their upregulation suggests alterations in skin architecture and potentially its mechanical properties. The Gene Ontology (GO) terms associated with these upregulated genes include skin development and epidermis development (Figure S1A–C), which are directly relevant to the observed morphological changes in the skin. The GO terms for the biological processes of these upregulated genes highlight several important functions: ribosome biogenesis, ribonucleoprotein complex biogenesis, skin development, and epidermis development (Figure S1B). These processes are essential for the growth and maintenance of skin cells, as well as for skin barrier formation and function. Intermediate filament binding was also identified as a significant molecular function GO term, which is critical for maintaining skin integrity (Figure S1A). Genes such as KRT14, NME1, EVPL, FLG, and FAM83H are involved in intermediate filament binding, contributing to the structural framework that maintains skin resilience. Further analysis through KEGG pathway analysis revealed that 10 upregulated genes are involved in the melanogenesis pathway, which is related to skin pigmentation and may play a role in skin response to stress or damage (Figure 2B). These findings suggest that rGLP-1 exposure may alter skin development at a molecular level, potentially affecting skin structure, pigmentation, and resilience.

#### 3.1.2. Molecular Changes in the Brain

**Figure 3:**
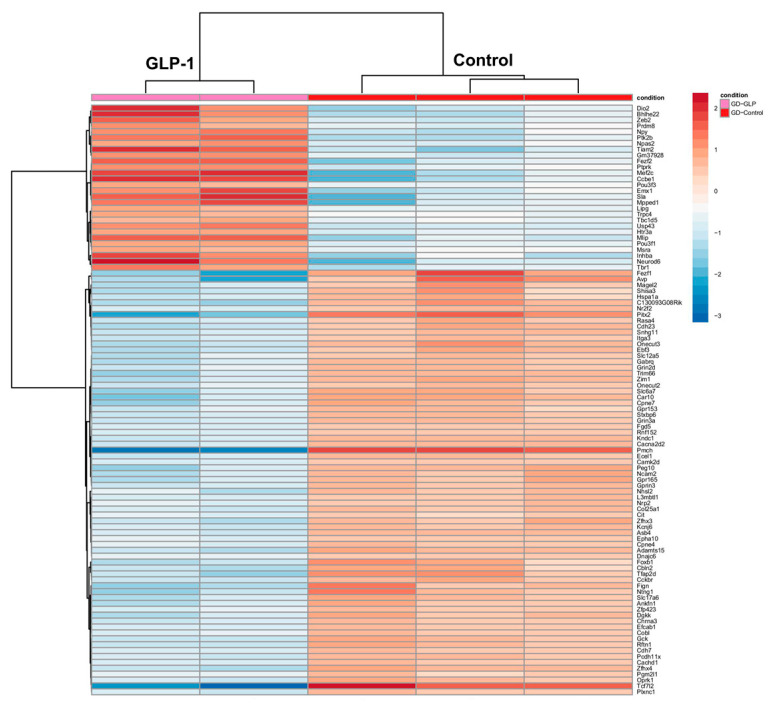
Differential gene expressions in the neonates’ brains born to rGLP-1 injected mice. The heatmap shows the differentially expressed genes in the brains of newborn pups born to a mother exposed to rGLP-1.

rGLP-1 injection in pregnant mice resulted in significant differential expression in the neonatal brain of their pups. A heatmap (Figure 3) displays the top 100 differentially expressed genes, with 28 genes upregulated (DIO2, ZEB2, BHLHE22, NEUROD6, PTPRK, INHBA, etc.) and 72 genes downregulated (FEZFL, AVP, PITX2, and ADAMTS15). Gene Ontology (GO) analysis identified 20 relevant GO terms spanning biological processes, cellular components, and molecular functions (Figures S2 and S3A). Key biological processes associated with upregulated genes included synapse organization, axonogenesis, forebrain development, and regulation of neurogenesis (Figure S3A). In terms of cellular components, significant changes were observed in the synaptic membrane, asymmetric synapse, neuron-to-neuron synapses, postsynaptic density, and postsynaptic specialization. For molecular functions, genes related to gated channel activity, ion channel activity, passive transmembrane transporter activity, and metal ion transmembrane transporter activity were notably altered. A KEGG pathway analysis revealed 20 critical pathways, with significant changes in neuroactive ligand-receptor interactions, the cAMP signaling pathway, the calcium signaling pathway, and axon guidance (Figure S3B).

#### 3.1.3. Molecular Changes in the Lung

**Figure 4:**
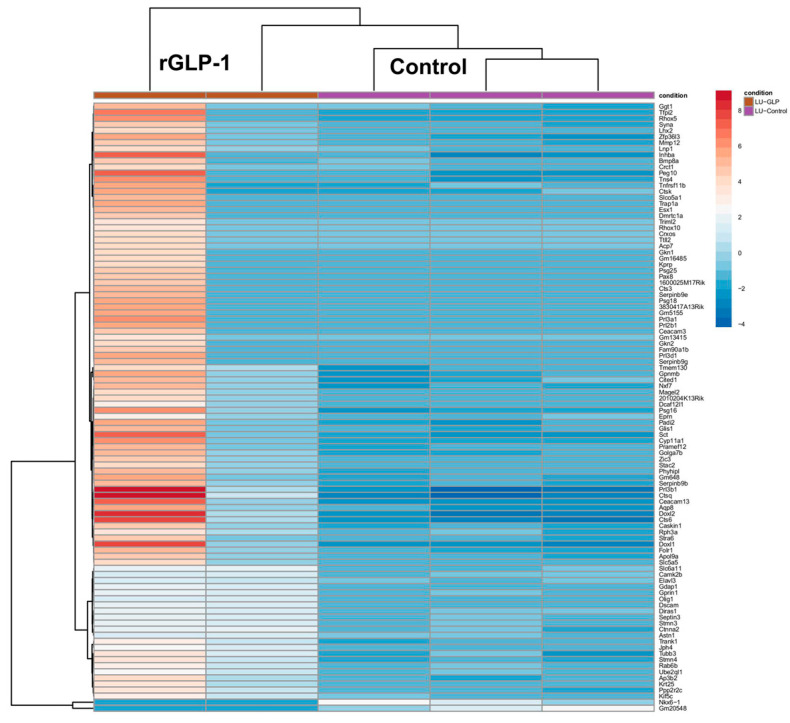
Differential gene expression profiles of the neonates’ lungs born to rGLP-1 injected mice. The heatmap shows 100 differentially expressed genes in the neonate lungs.

The lungs of GLP-1-exposed mouse pups displayed significant differential expression of 2463 genes, with a heatmap illustrating the top 100 differentially expressed genes in lung tissue (Figure 4). Upregulated genes included INHBA, PEG10, CEACAM13, PR13A1, AQP8, DOX12, PR1361, DOX1, CTS9 and CTS6 while NKX6-1 and Gm20548 were downregulated. Notably, INHBA is a well-known tumor promoter under tumor-bearing conditions, and AQP8 is upregulated under inflammatory conditions, highlighting their potential roles in lung pathology. Gene Ontology (GO) analysis identified 20 relevant GO terms, with key biological processes including gland development, regulation of body fluid levels, regulation of epithelial cell proliferation, and positive/negative regulation of secretion. Molecular functions associated with these changes included metal ion transmembrane transporter activity. Notable cellular components included cation channel complexes and exocytic vesicles. The KEGG pathway analysis highlighted 11 significant pathways, with genes involved in neuroactive ligand-receptor interactions and Ras signaling pathways in the lung (Figure S4).

#### 3.1.4. Molecular Changes in the Liver

**Figure 5:**
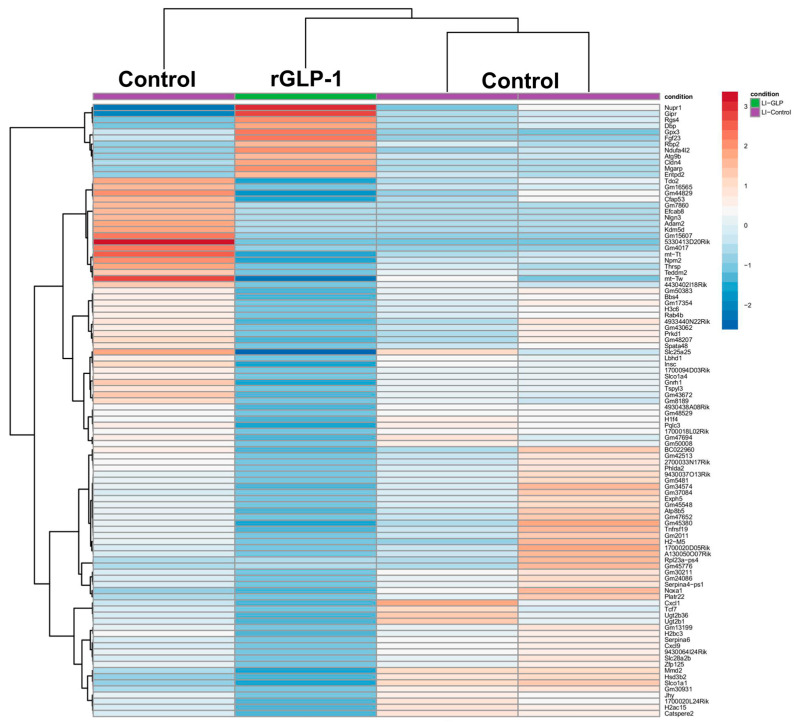
Differential gene expression profiles of the neonates’ livers born to rGLP-1 injected mice. The heatmap shows differentially expressed genes in the liver of pups born to rGLP-1 injected mice.

The hepatic tissue of GLP-1 mouse pups shows 682 significantly differentially expressed genes. The heatmap reveals 100 significantly differentially expressed liver genes (Figure 5). Notably, NUPR1, RGS4, ATG9B, GPX3, RBP2, ENTPD2, and CLDN4 were upregulated in pup livers, while TDO2, SLC25A25, MT-TT, NPMM2, MMD2, SLC0191, CXCL1, and CXCL9 were downregulated. The GO analysis shows 5 biological processes, 5 cellular components, and 20 molecular functions related to the liver. Significant biological processes in the liver include steroid metabolism, carboxylic acid biosynthesis, and organic acid biosynthesis. Transporter complexes and transmembrane transporter complexes were identified as key cellular components. For molecular functions, NADH dehydrogenase activity and active transmembrane oxidoreductase activity were notably altered. The KEGG pathway analysis highlighted 13 distinct pathways, including chemical carcinogenesis-DNA adducts, chemical carcinogenesis-reactive oxygen species, and receptor interactions critical for liver function (Figure S5).

### 3.2. Real-Time PCR Analysis

**Figure 6:**
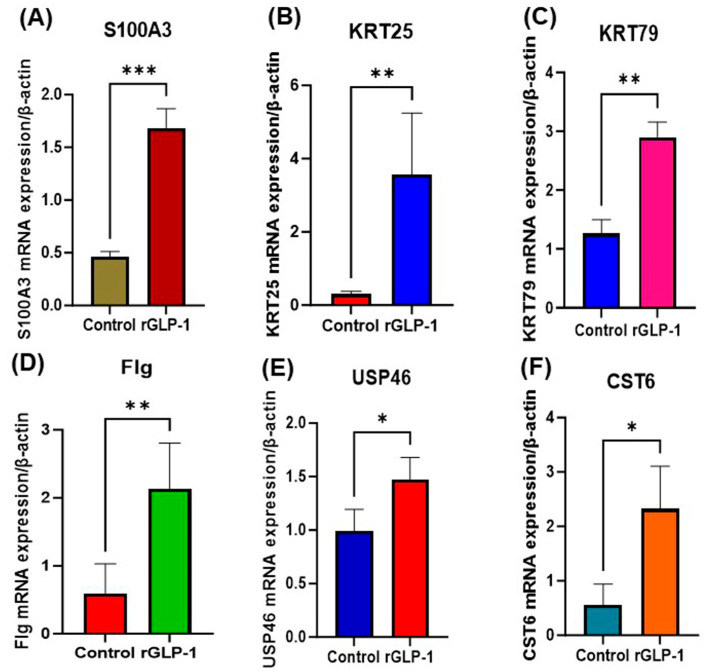
Significantly altered mRNA expressions in the skin of pups born to GLP-1-ex posed mothers. (**A**) S100A3 mRNA expression compared to the control. (**B**) KRT25 mRNA expression compared to the control. (**C**) KRT79 mRNA expression compared to the control. (**D**) Flag mRNA expression compared to the control. (**E**) USP46 mRNA expression compared to the control. (**F**) CST6 mRNA expression compared to the control. * Statistically significant. *** = *p* < 0.0005; ** = *p* < 0.005; * = *p* < 0.05.

The RT-PCR analysis revealed the significantly increased expression of S100A3, KRT25, KRT79, FLG, USP46, and CST6 at the mRNA level (Figure 6A–F). These results confirm the RNA sequencing analysis of the skin.

## 4. Discussion

Medications taken during pregnancy have the potential to cross the placenta and directly affect fetal development through various mechanisms such as oxidative stress, epigenetic modifications, metabolic activation, or indirectly through placental dysfunction [12]. Hormonal changes triggered by drugs can disrupt normal growth and development, particularly in sensitive tissues like the brain [13]. One class of medications gaining popularity for their role in treating obesity and type 2 diabetes is GLP-1 receptor agonists (GLP-1RAs), which mimic the action of the naturally occurring incretin hormone GLP-1. Despite their therapeutic benefits, the safety of GLP-1RA use during pregnancy remains under scrutiny, as these medications have been linked to both beneficial and harmful effects on fetal development.

Our study found that GLP-1 exposure in pregnant mice led to increased fetal mortality, a reduction in both maternal and pup weight, and morphological changes in the skin of the pups. These results were accompanied by significant alterations in gene expression across multiple tissues, including the skin, brain, lungs, and liver, as revealed by mRNA sequencing. All tissue samples exhibited changes in gene expression, providing insights into the molecular disruptions caused by GLP-1 exposure. In the skin, upregulated expression of keratin (KRT) genes, including KRT79, KRT75, and KRT71, points to abnormal skin development that could contribute to various dermatological disorders. While in the brain, upregulation of genes like DIO2 suggests disruptions in thyroid signaling, which could impair neuronal development and function. These findings underscore the complexity of maternal GLP-1 exposure and the potential for neonatal mortality and long-term health complications.

### 4.1. Skin

Skin GLP-1 and its receptor (GLP-1R) are important in skin development and folliculogenesis. In our study, GLP-1 exposure led to the upregulation of several KRT genes, which are essential for the formation and integrity of the epidermis and hair follicles [14]. These genes play key roles in the structural formation of epithelial tissues, with mutations often leading to keratopathies. Specifically, KRT75, KRT71, and KRT25 (which were found to be increased in both gene sequencing and qPCR analysis) are involved in hair and nail development, with mutations in these genes linked to conditions such as wooly hair and hypotrichosis [15]. Overexpression of S100a3 (which is found to be increased in both gene sequencing and qPCR analysis), a protein involved in keratinocyte maturation, further supports the idea that GLP-1 exposure disturbs normal skin formation. Moreover, genes like DICER1, ACOT1, and CST6 (increased in both gene sequencing and qPCR analysis), involved in hair follicle formation and skin barrier integrity, were also upregulated, suggesting potential skin abnormalities. Interestingly, some downregulated genes, such as SLC16A2 and SLC9A6, which are associated with skin differentiation, further suggest that GLP-1 exposure may hinder proper skin development. These molecular alterations indicate that GLP-1 may contribute to skin defects and impair epidermal function, making pups more vulnerable to environmental stressors.

### 4.2. Brain

Our findings revealed notable changes in brain development following rGLP-1 exposure. Our mRNA sequencing results show an upregulation of genes like BHLHE22 and NEUROD6, which play important roles in neurogenesis and neuronal survival. BHLHE22 is a transcription factor involved in regulating body weight, energy homeostasis, and fertility [16]. Its upregulation suggests a potential compensatory response to altered metabolic signaling in GLP-1-exposed pups. NEUROD6, a gene essential for neuronal differentiation, also showed increased expression, which 13 may indicate adaptive responses to stress. These findings suggest that while GLP-1 may promote neurogenesis under certain conditions, it could also disrupt normal neuronal function. On the other hand, genes such as DIO2 and ZEB2 were overexpressed, which points to potential disruptions in thyroid hormone signaling and neurodevelopmental processes. DIO2, which encodes an enzyme involved in thyroid hormone metabolism, has been associated with abnormal brain development and behavioral changes when overexpressed [17]. Similarly, ZEB2, a gene linked to neurodevelopmental disorders like Mowat–Wilson syndrome, could contribute to abnormalities in brain formation [18]. These molecular changes suggest that GLP-1 exposure during pregnancy may negatively impact neurodevelopment, potentially leading to cognitive and behavioral disorders in offspring.

### 4.3. Liver

The liver is another organ affected by GLP-1 exposure. Several genes associated with metabolic regulation and stress response, like NUPR1, RGS4, and ATG9B, were upregulated in the liver of GLP-1-exposed pups. NUPR1 plays a protective role in the liver by responding to metabolic stress, but its overexpression has been linked to tumorigenesis [19]. The downregulation of genes like SLC25A5/ANT2, involved in mitochondrial function, suggests that disruptions in cellular metabolism may also affect liver function and contribute to blood development failure in pups [20].

### 4.4. Lungs

The lungs of GLP-1-exposed pups also exhibited significant molecular changes. Upregulation of genes from the prolactin (PRL) family, such as PRL2B1 and PRL3A1, could indicate an adaptive response to enhance tissue repair and survival in the lungs [21]. Similarly, serpins (serpineb9b, serpineb9e, and serpineb9g), a family of protease inhibitors involved in immune regulation, were 14 also upregulated, but their precise role in lung pathology remains unclear. ZFP36L3, a gene linked to neonatal survival, was overexpressed, which may reflect an attempt to increase stress resistance but could also be detrimental to lung function in the long term [22]. Additionally, INHBA (Inhibin—B-A), a gene associated with lung cancer progression, was upregulated, which may raise concerns about the potential carcinogenic effects of GLP-1 exposure on the lungs [23]. This study provides valuable insights into the molecular mechanisms that underline the effects of GLP-1 exposure during pregnancy on fetal development. The upregulation of genes associated with skin abnormalities, neurodevelopmental disruptions, liver dysfunction, and lung pathology suggests that maternal GLP-1 exposure may lead to significant adverse outcomes for offspring health. These findings underscore the importance of carefully considering the risks of GLP-1RA use during pregnancy, particularly during critical periods of fetal development. However, the reliance on a murine model limits the direct translation of these results to human physiology, and further functional validation is necessary to confirm these findings. Overall, this research highlights the need for ongoing investigation into the long-term consequences of GLP-1 exposure to maternal and fetal health, particularly as the use of GLP-1 medications continues to rise.

### 4.5. Strengths and Limitations

A key strength of this study is the use of a carefully controlled experimental model to assess the effects of rGLP-1 exposure during specific stages of early and late pregnancy, as shown by previous research [9,10]. This method enabled the pinpointing of timing-specific impacts on maternal and fetal outcomes. Including RNA sequencing in neonates allowed for a detailed analysis of gene expression changes across various tissues, offering new insights into the molecular mechanisms behind the phenotypic abnormalities caused by GLP-1 exposure during pregnancy, which had not been shown before. Importantly, using recombinant GLP-1 agonists improved the study by isolating the specific effects of synthetic GLP-1, removing confounding effects linked to the clinical formulations of the peptide.

Although the study has strengths, it also has several limitations. Research on GLP-1 use in humans, especially during pregnancy, is limited, although some cases of its use during pregnancy have been reported recently [2,3,4]. It is possible that some subjects used GLP-1 one or more times before pregnancy, which was not considered here due to the scarcity of literature on GLP-1 use, including the frequency, doses, and timing of administration during pregnancy in humans. Additionally, a single injection has shown some effects in our initial studies. The changes we observed might have been underestimated.

## 5. Conclusions

This study highlights the potential risks associated with GLP-1 receptor agonist (GLP-1RA) exposure during pregnancy, particularly in later stages. Maternal weight loss increased neonatal mortality, and skin abnormalities in pups were observed in pups following rGLP-1 exposure. mRNA sequencing revealed significant changes in gene expression across multiple organs, including the skin, liver, brain, and lungs, suggesting that rGLP-1 exposure disrupts normal developmental processes. In the skin, the upregulation of keratin-related genes points to potential structural changes, which were apparent in pups. These findings suggest alterations in skin 15 architecture and mechanical properties, which could contribute to dermatological abnormalities. In the brain, changes in neurogenesis-related genes like BHLHE22 and Neurod6 indicate potential disruptions in neuronal development. The liver and lungs also exhibited significant molecular changes, suggesting disturbances in metabolic and immune pathways. These results highlight the need for caution regarding the use of GLP-1RAs during pregnancy, especially in later gestation. This research provides valuable insights into the molecular mechanisms behind the observed maternal and fetal complications and underscores the importance of further investigation into the long-term effects of GLP-1 exposure.

Nonetheless, we believe this study strongly indicates that pregnant women should exercise caution when using GLP-1. Therefore, a comprehensive study examining the effects of GLP-1 exposure at various time points with different doses, both before and during pregnancy (despite limited human data), will be conducted in the future.

## Figures and Tables

**Table 1:** Primers used in the RT-PCR analysis.

Gene Name	Gene Description	Primer Sequence
CST6	Cystatin 6	GAACTTGTCACCCACCGACC TTTGGTGTCTCGGAAGTAGTAGA
KRT25	Keratin 25	CAACGCCAACGCTGTACTG ACGTCAGCCTCTACGCTCT
Flg	Flaggrin	ATGTCCGCTCTCCTGGAAAG TGGATTCTTCAAGACTGCCTGTA
RPs19	Ribosomal Protein S19	CAGCAGGAGTTCGTCAGAGC CACCCATTCGGGGACTTTCA
Krt 79	Keratin 79	GTCCAGGCAGACTTTCTCCAC ACATAGTCACTGAGCTGTAGCTG
Krt 35	Keratin 35	ATGACTGTCCGAAACATCGCC TTGACCAATCCGAAGTAGTGTTC
USP46	Ubiquitin specific peptidase 46	ATGACTGTCCGAAACATCGCC TTGACCAATCCGAAGTAGTGTTC
S100a3	S100 calcium binding domain a3	CAGTAGCTGCCATCGTGTG TACTCCCCAAAGTCCACTTCG
B-Actin	HouseKeeping gene	ATGACCCAAGCCGAGAAGG CGGCCAAGTCTTAGAGTTGTTG

## Data Availability

The raw data supporting the conclusions of this article will be made available by the authors on request.

## Supplementary Materials

The following supporting information can be downloaded at: https://www.mdpi.com/article/10.3390/jdb13030029/s1, Figure S1: Go analysis of skin from pups born to rGLP-1-exposed mother; Figure S2: GO terms related to DEGs of the Brain; Figure S3: Differential gene expressions in the neonate brain born to rGLP-1 injected 449 mice; Figure S4: The KEGG pathway analysis highlighted 11 significant pathways in the lung of GLP-1 468 exposed mouse pups; Figure S5: The KEGG analysis shows the pathways related to the liver of differentially expressed genes of the neonate liver born to rGLP-1 injected mice.

## Abbreviations

The following abbreviations are used in this manuscript:

EP	Early Pregnancy
LP	Late Pregnancy
GLP-1	Glucagon-like peptide-1
KEGG	Kyoto Encyclopedia of Genes and Genomes
GO	Gene Ontologies
GLP-1Ras	Glucagon-like peptide-1 receptor agonists
PCOS	Polycystic ovary syndrome
PBS	Phosphate-buffered saline
rGLP-1	recombinant GLP-1
RNA	Ribonucleic acid
mRNA	messenger RNA
