# Supplementary material for: GLP-1-Mediated Pregnancy and Neonatal Complications in Mice
Source: J Dev Biol. 2025 Aug 15;13(3):29. doi: 10.3390/jdb13030029 (PMC12372104; doi:10.3390/jdb13030029)
Supplement: Supplementary file 1 [file jdb-13-00029-s001.zip › jdb-3667680-supplementary.pdf]

Supplementary Figures

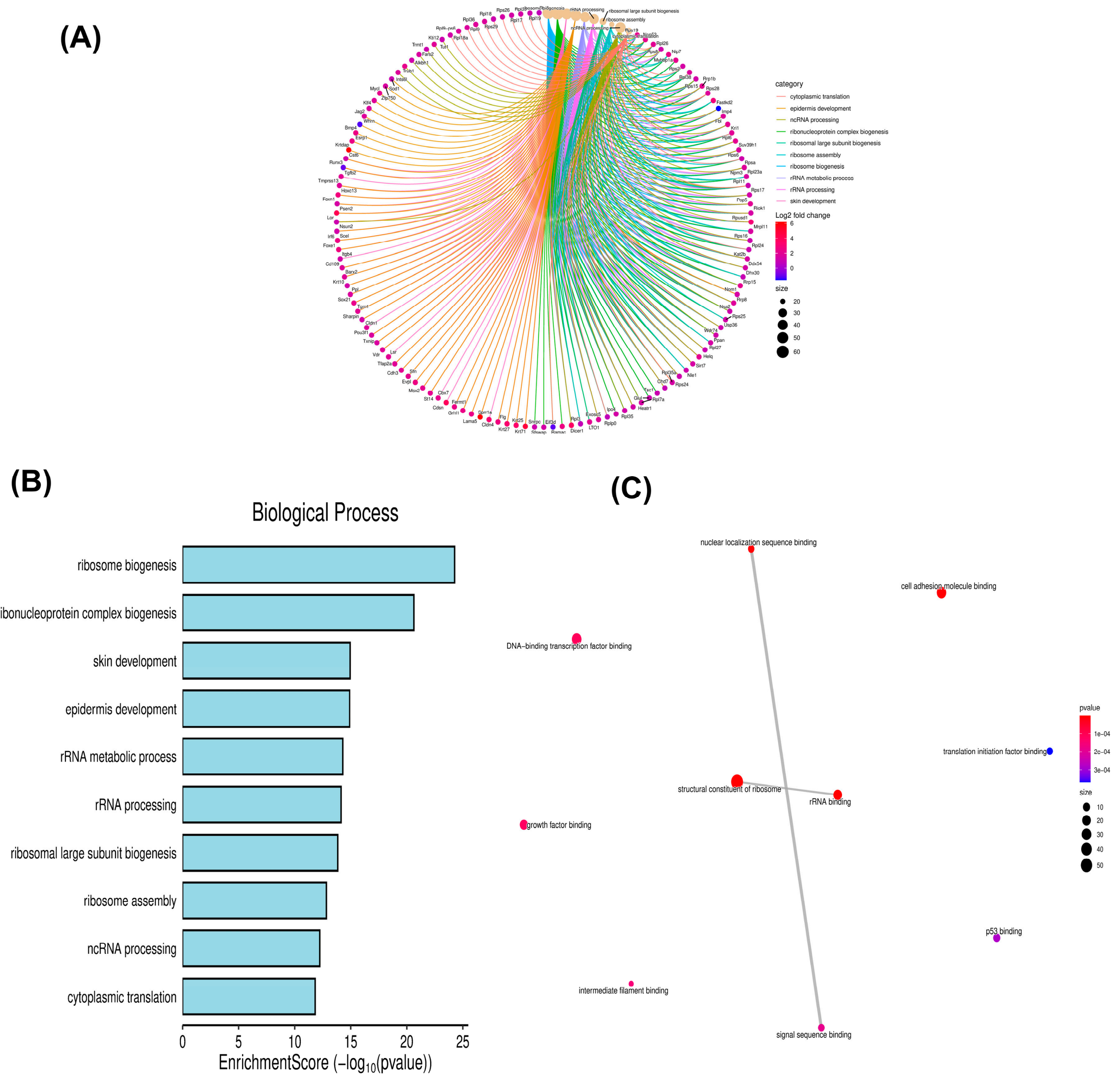

Figure S1. **Go analysis of skin from pups born to rGLP-1-exposed mother.** (**A**) The cnet plot shows genes involved in skin development and epidermis development. (**B**) The bar plot shows the GO terms related to the biological process. (**C**) The emap plot shows the significant GO terms related to skin, intermediate filament binding.

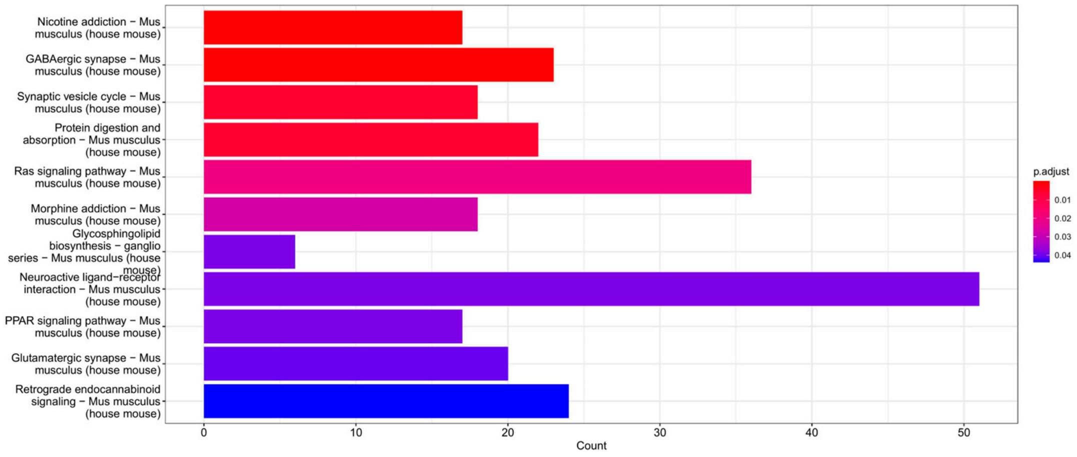

Figure S2: GO terms related to DEGs of the Brain. A bar plot shows the cellular component.

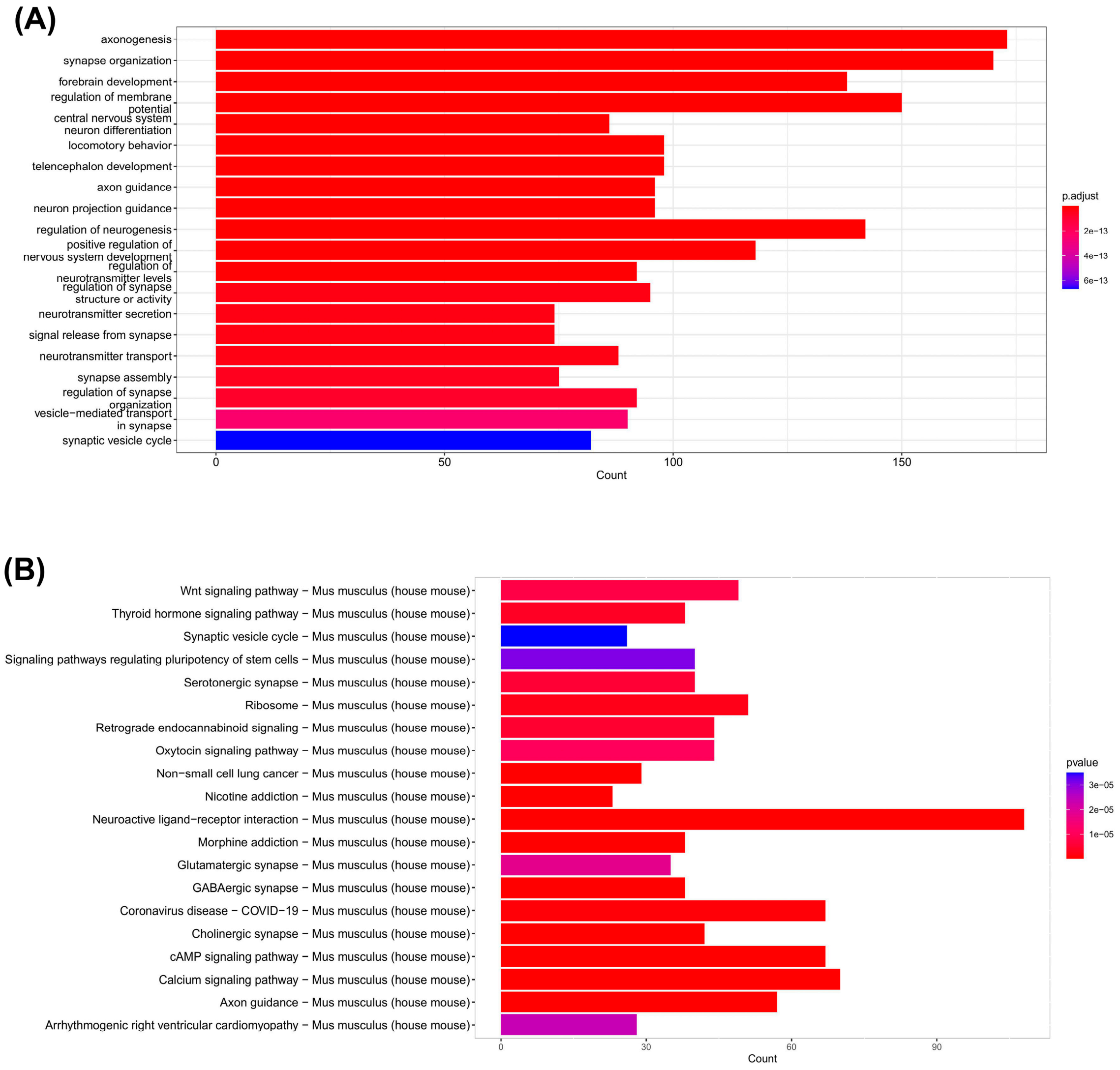

Figure S3. Differential gene expressions in the neonate brain born to rGLP-1 injected 449 mice. (A) The bar plot shows the GO terms for biological processes related to the brain 450 are forebrain development, synapse assembly, and axon guidance. (B) The bar plot of 451 KEGG analysis shows different affected pathways in the brain, like glutamatergic and 452 GABAergic synapses and calcium signaling pathways.

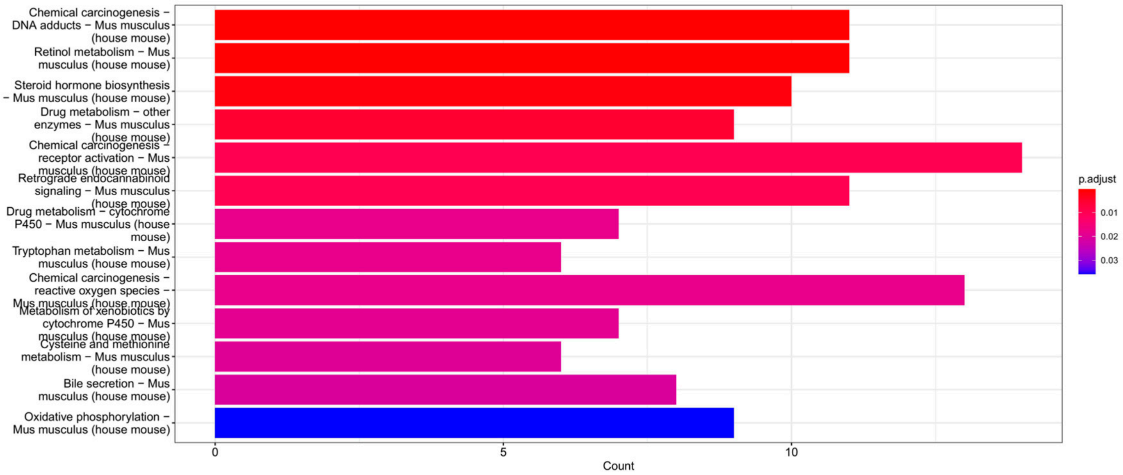

Figure S4. The KEGG pathway analysis highlighted 11 significant pathways in the lung of GLP-1 468 exposed mouse pups.

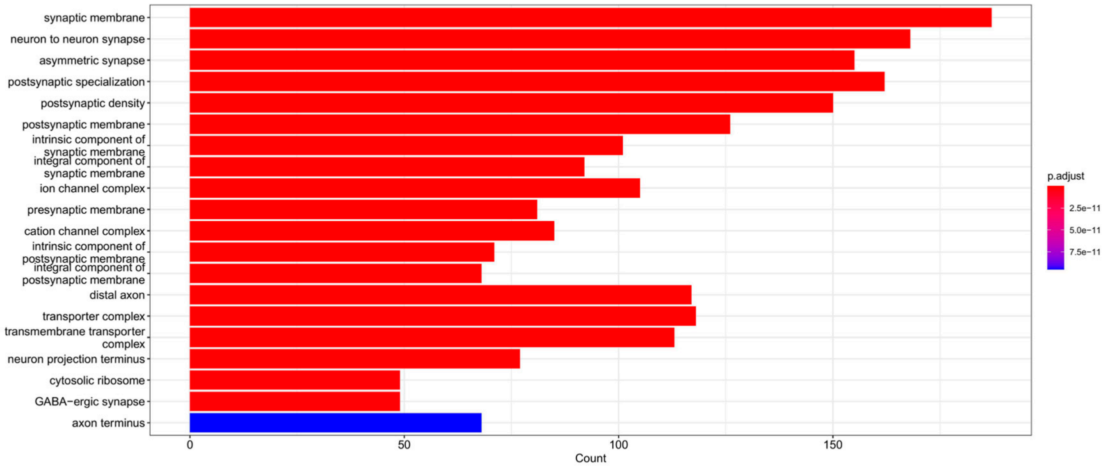

Figure S5. The KEGG analysis shows the pathways related to the liver of differentially expressed genes of the neonate liver born to rGLP-1 injected mice.
